# The conundrum of the Epstein-Barr virus-associated gastric carcinoma in the Americas

**DOI:** 10.18632/oncotarget.18497

**Published:** 2017-06-15

**Authors:** Gonzalo Carrasco-Avino, Ismael Riquelme, Oslando Padilla, Miguel Villaseca, Francisco R. Aguayo, Alejandro H. Corvalan

**Affiliations:** ^1^ Advanced Center for Chronic Diseases (ACCDIS), Pontificia Universidad Catolica de Chile, Santiago, Chile; ^2^ Department of Pathology, Faculty of Medicine, Universidad de Chile, Santiago, Chile; ^3^ Scientific and Technological Bioresource Nucleus (BIOREN), Universidad de la Frontera, Temuco, Chile; ^4^ Department of Pathology, Universidad de la Frontera, Temuco, Chile; ^5^ Department of Public Health, School of Medicine, Pontificia Universidad Catolica de Chile, Santiago, Chile; ^6^ Department of Basic and Clinical Oncology, Faculty of Medicine, Universidad de Chile, Santiago, Chile; ^7^ UC-Center for Investigational Oncology (CITO), Pontificia Universidad Catolica de Chile, Santiago, Chile; ^8^ Department of Hematology and Oncology, School of Medicine, Pontificia Universidad Catolica de Chile, Santiago, Chile

**Keywords:** gastric cancer, Epstein-Barr virus, americas, molecular classification, phylogeographic diversity

## Abstract

Epstein-Barr virus-associated gastric carcinoma shows a higher prevalence in the Americas than Asia. We summarize all studies of Epstein Barr virus-associated gastric carcinoma in the Americas, focusing on host characteristics, environmental associations and phylogeographic diversity of Epstein-Barr virus strains. In the Americas, the prevalence of Epstein Barr virus-associated gastric carcinoma is 11.4%, more frequent in males and portray predominantly diffuse-type histology. EBERs, EBNAs, BARTs and LMP are the highest expressed genes; their variations in healthy individuals may explain the phylogeographic diversity of Epstein-Barr virus across the region. Gastric cancer cases harbor exclusively the western genotype (subtype D and kept Xho I site), suggesting a disrupted co-evolution between the pathogen and its host. Epstein-Barr virus-associated gastric carcinoma molecular subtype cases from The Cancer Genome Atlas display PIK3CA gene mutations, amplification of JAK2, PD-L1 and PD-L2 and CpG island methylator phenotype, leading to more extensive methylation of host and viral genomes than any other subtypes from the study. Environmental conditions include negative- and positive- associations with being firstborn child and smoking, respectively. A marginal association with H. pylori has also been reported. Lymphoepithelioma-like carcinoma is associated with Epstein Barr virus in 80%–86% of cases, most of which have been included as part of Epstein Barr virus-associated gastric carcinoma series (prevalence 1.1%–7.6%). Whether these cases represent a variant of Epstein-Barr virus-associated gastric carcinoma is discussed. We propose novel research strategies to solve the conundrum of the high prevalence of Epstein-Barr virus-associated gastric carcinoma in the Americas.

## INTRODUCTION

Gastric cancer is the fifth most common malignancy and the third leading cause of cancer death in both sexes worldwide, accounting for 951,000 new cases and 723,000 deaths in 2012 [1]. As with other infection-related cancers, an increasing proportion of gastric cancer falls on low- and middle-income countries including the Americas-particularly, Central and South America [2]. The highest prevalence rates have been observed in countries from the pacific coast such as Chile, Costa Rica, Colombia, and Ecuador (i.e. > 20 cases per 100 000 men); whereas the lowest rates have been observed in the U.S., Canada, Argentina, Cuba, and Puerto Rico (< 4/100000) [2]. Due to the growth and aging of the population, gastric cancer is expected to escalate from the fifteenth to the tenth cause of death in all-cause mortality within the region in the next 15 years [3].

In this scenario, novel molecular classifications and meta-analyses have identified Epstein-Barr virus (EBV) as a distinct etiological agent for gastric cancer [4–7]. A striking feature of EBV-associated gastric carcinoma (EBVaGC) is the high prevalence observed in the Americas as compared with Asia (OR: 1.7, 95% CI:1.1–2.7) [7]. The main purposes of this review are to describe the clinical, molecular and environmental characteristics of EBVaGC in the Americas, and to propose novel research strategies for the exploration of this conundrum.

### EBV-associated gastric carcinoma in the **Americas**

**Figure 1:**
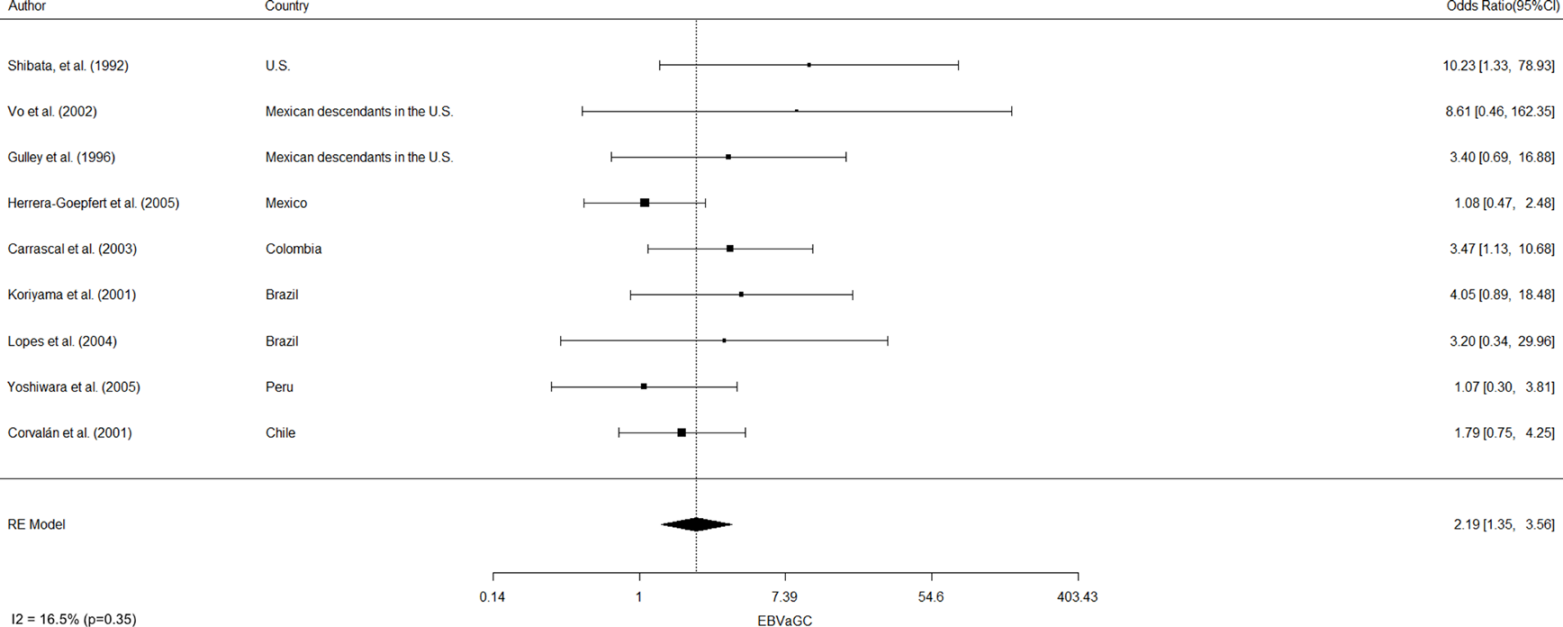
Estimated odds ratio (95% CI) for male predominance of EBV-associated gastric cancer in the Americas Meta-analyses were performed by a random-effects model with the Inverse variance method, using the DerSimonian-Laird estimator, a logit transformation and the Clopper-Pearson confidence interval for individual studies. The results are shown in a log-scale.

**Figure 2:**
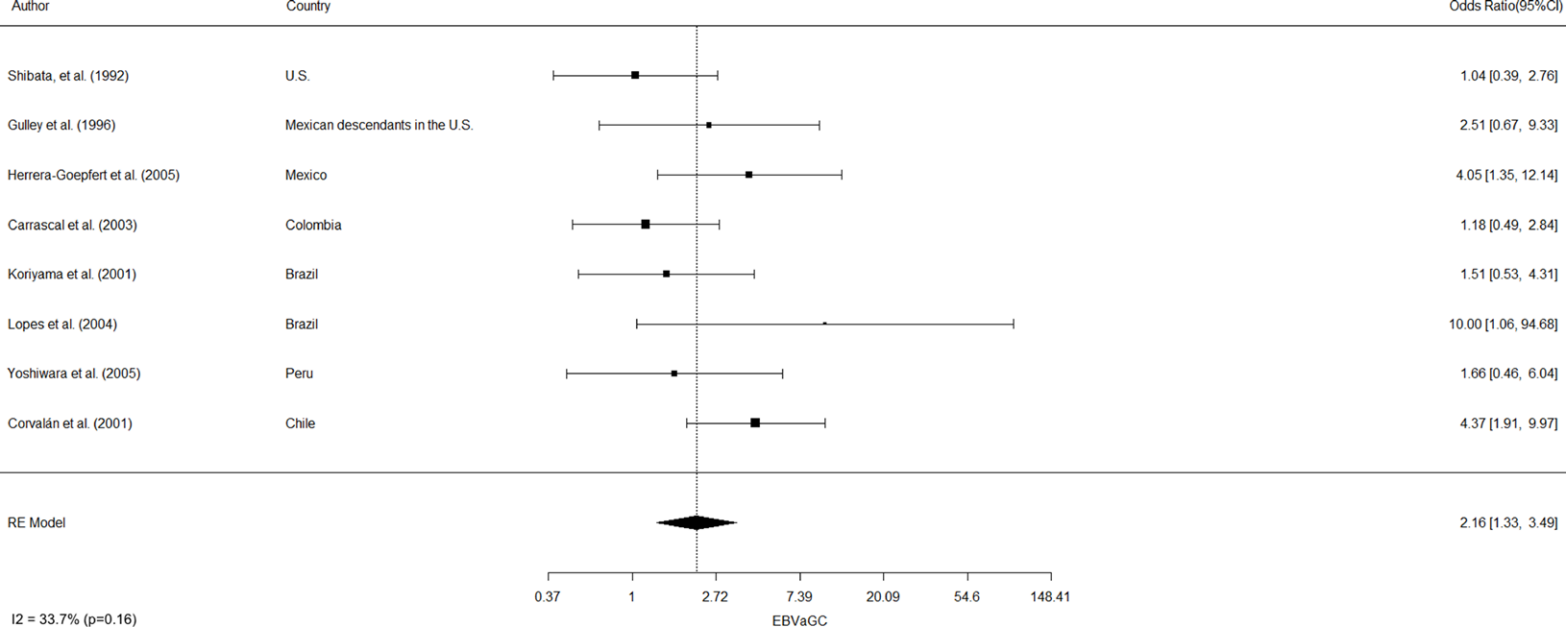
Estimated odds ratio (95% CI) for diffuse-type histology of EBV-associated gastric carcinoma in the Americas Meta-analyses were performed by a random-effects model with the Inverse variance method, using the DerSimonian-Laird estimator, a logit transformation and the Clopper-Pearson confidence interval for individual studies. The results are shown in a log-scale.

EBVaGC in the Americas was first reported by Shibata and coworkers [8]. These authors identified 22 of 138 (16%) EBV-positive cases in U.S. GC cases by *in-situ* hybridization of EBV-encoded small RNAs (EBERs) in tumor samples. In positive cases, EBERs were expressed exclusively in gastric tumor cells. No EBV sequences were detected in the surrounding lymphocytes or precancerous lesions, such as chronic gastritis or intestinal metaplasia. In addition, EBVaGCs were more frequently observed in males than females (*p* = 0.007). Subsequent studies by Gulley and coworkers [9] and Vo and coworkers [10], identified 11 of 95 (12%) and 9 of 43 (20%) EBV-positive GC cases, respectively, in Mexican descendants living in the U.S. However, only Vo et al. [10] confirmed male predominance (*p* = 0.01). Later, Herrera-Goepfert et al. [11] identified 24 of 330 (7.3%) EBV-positive cases in Mexico City. In this series, no male predominance was confirmed. Interestingly, all cases were of diffuse-type histology. In Colombia, Carrascal et al., [12] examined 178 consecutive gastric carcinoma cases identifying 23 (13%) cases of EBVaGCs. In this study, EBVaGCs were most often detected in males (*p* = 0.004), and located predominantly on the non-antrum stomach (*p =* 0.009). Koriyama et al., [13] examined 151 cases from Brazil, detecting 11.2% of EBVaGC. Although EBVaGC was most frequent among males (*p* = 0.047), Lopes and coworkers [14] did not confirm this finding in a posterior study. Another series consisting of 254 gastric cancer cases from Japanese descendants living in Peru were evaluated by Yoshiwara et al., [15], identifying only 3.9% EBVaGC (10 cases). In this series, no evident predominance by gender or histological subtype were detected. Finally, in Chile, Corvalan and coworkers [16] detected 31 of 185 (16.8%) EBV-positive gastric cancer cases. In this series, predominance of diffuse-type histology (*p <* 0.001) and non-antrum location (*p =* 0.01) were the most significant findings. No reports of EBVaGC have been described in other American countries. A consolidated overview of EBVaGC in the Americas is shown in Table 1. The meta-analytic estimated prevalence of EBV positivity in the region was 11.49% (95% CI = 8.46 to 15.43), with high heterogeneity among studies (I^2^ = 73.3%; *p <* 0.001). Interestingly, heterogeneity for predominant sex, location and histology was low (I2=16.5% [*p =* 0.35]; 0% [*p =* 0.68] and 33.7% [*p =* 0.16], respectively). Univariate analysis shows male predominance (*p <* 0.001), non-antrum location (*p <* 0.001) and diffuse-type histology (*p <* 0.001) as the most significant features of EBVaGC in the Americas. Odds-ratio estimated for male predominance and diffuse histology were 2.19 (95% CI: 1.35–3.56) and 2.16 (95% CI: 1.33–3.49), respectively (see Figure 1 and Figure 2).

**Table 1:** EBV-associated gastric cancer in Latin America

			Sex			Histology			Location		
Authors	Country	Frequency	Male	Female	*p* value	Intestinal	Diffuse	*p* value	Antrum	Non-antrum	*p* value
Shibata et al. (1992)	U.S.	22/138 (16%)	21/99	1/39	0.007049	15/95	7/43	1	ND	ND	ND
Vo et al. (2002)	Mexican descendants in the U.S.	9/43 (20.9%)	9/32	0/11	0.012667	ND	ND	ND	ND	ND	ND
Gulley et al. (1996)	Mexican descendants in the U.S.	11/95 (12%)	9/50	2/33	0.116414	4/47	7/37	0.160373	ND	ND	ND
Herrera-Goepfert et al. (2005)	Mexico	24/330 (7.3%)	13/173	11/157	0.859116	4/141	20/189	0.007012	16/192	8/156	0.24061
Carrascal et al .2003	Colombia	23/178 (13%)	19/108	4/69	0.022833	11/91	12/86	0.712179	14/67	6/81	0.016888
Koriyama et al. (2001)	Brazil	17/151 (11.2%)	15/102	2/49	0.05314	6/66	11/84	0.442513	3/11	8/95	0.052271
Lopes et al. (2004)	Brazil	6/53 (11.3%)	5/30	1/17	0.287087	1/27	5/18	0.019945	1/6	2/22	0.594853
Yoshiwara et al. (2005)	Peru	10/254 (3.9%)	5/115	5/123	1	4/127	6/117	1			
Corvalán et al. (2001)	Chile	31/185 (16.8%)	23/121	8/69	0.183499	10/114	21/71	< 0.001	25/119	4/63	0.01015
	Consolidate	153/1427 (10.7%)	119/830	34/567	< 0.001	40/613	82/602	< 0.001	59/395	28/417	< 0.001

The meta-analytic estimated prevalence of EBV positivity in the region was 11.49% (95% CI = 8.46 to 15.43).

Meta-analyses was performed by a random-effects model with the Inverse variance method, using the DerSimonian-Laird estimator, an logit transformation and the Clopper-Pearson confidence interval for individual studies.

I2 = 73.3% (*p* < 0.001).

### Role of viral factors in the pathogenesis of EBV-associated gastric carcinoma

#### Overview of viral machinery of EBV

EBV is a 175Kbp double-stranded lineal DNA virus that belongs to the gamma herpesvirus family [17]. The main feature of these viruses is the establishment of persistent latent infections and the possibility of viral reactivation under determined conditions. Thus, the virus persists throughout life without any possibility of clearance [17]. The sequence of events leading to a latent infection are: i) virus entry to cells that express the CD21 receptor, ii) virus transportation to the nucleus, iii) genome circularization and chromatinization, iv) transcription of latency-associated genes and v) repression of lytic cycle genes [18]. In the neoplastic process, the virus remains in a latent stage in every tumor cell, repressing the expression of genes involved in viral replication. However, virus integration into the host genome almost never occurs, and the EBV genome is always found in an episomal form within the cell nucleus. However, there is one report in The Cancer Genome Atlas (TCGA) network showing one gastric cancer case with evidence of integration of the EBV genome into the human genome, in which multiple independent RNAseq reads revealed a fusion transcript predicted to join the first 20 amino acids of the human plasminogen receptor (PLGRKT, alias C9orf46) to almost the entire coding sequence of the early lytic EBV gene BHLF1 (alias EA-D) [19]. The expression patterns of some specific EBV genes define the type of latency into the cells (for review see [20]). In EBVaGC the virus shows a type I/II latency, where the most relevant genes associated with the pathogenesis are: EBV encoded RNAs (EBERs), the EBV Nuclear Antigens (EBNAs), the BamH1-A rightward transcripts (BARTs) and, apparently, some Latent Membrane proteins (LMP) genes are actively expressed [17]. The EBER-1 and -2 genes are the most abundant small non-coding RNAs that bind to host proteins and are generally used as a target for EBV detection by *in situ* hybridization (ISH) [21]. The EBNA-1 and -2 genes are exclusively nuclear proteins expressed in latent infected gastric carcinoma cells, and related with the disruption of promyelocytic leukemia nuclear bodies [22]. EBNA-1 is a DNA binding protein that lacks enzymatic activity, although is able to bind with some cellular proteins, such as CK2 and possibly P32/TAP [23]. In addition, an important partner of EBNA-1 is the ubiquitin-specific protease USP7, which is able to stabilize p53 and mdm2 [24]. Interestingly EBNA-1 is expressed in all of the EBV-associated tumors and is involved in viral DNA replication, mitotic segregation and transcriptional activation [25]. BART genes are highly expressed and multispliced RNAs whose protein-coding function is highly controversial [26]. Although some BARTs open reading frames (ORFs) have been predicted, it remains unclear whether any of them can be endogenously translated. In addition, BART small and long non-coding RNAs are highly expressed and associated with oncogenic transformation and immune evasion functions [27, 28] (for review see [17, 20]). The LMP-1 and -2 genes encode for transmembrane proteins, with a plethora of oncogenic functions with conflicting results in gastric carcinoma [29, 30]. Interestingly, variations in its sequences might be associated with phylogeographic diversity of EBV strains in EBVaGC throughout the world [31, 32]. Taken together, EBV latent genes not only define the type of latency, but are associated with oncogenic transformation, immune evasion, and the genetic diversity of EBVaGC.

### EBV strains and EBV-associated gastric carcinoma

Previous characterization of restriction fragment length polymorphisms (RFLP) and the recently completed genome sequencing of 31 viruses lead towards the first global assessment of the genetic diversity of EBV. Twenty-five of these sequences were obtained from viruses present in malignant tumors, including 9 EBVaGC [33] and 7 cases which were isolated from benign lesions or healthy individuals [34–37]. Based on these data, five specific types (A, B, C, D and F) of EBV have been identified. Types A and B are defined by the substitution of 1.8 kb in the C-terminal domain of the EBNA-2 gene [38]. Subtype A is the predominant strain in Western and Asian countries, whereas subtype B is frequently found in Africa [38, 39]. In addition, these two types of EBV differ in their capacity to transform B-lymphocytes to a proliferative state [40]. RFLPs at the BamHI W1/I1 boundary region identify subtypes C and D. Type C lacks the BamHI site and is more frequent in Asia among healthy individuals and EBV-associated diseases [41–44]. Conversely, subtype D (which has retained the BamHI restriction site) prevails in Western countries [41, 45]. Another polymorphism at the BamHI site defines the F type, of which the prototype BamHI F virus is found worldwide, but the ‘‘f’’ variant, characterized by the presence of an extra BamHI site, is found only in cases of nasopharyngeal carcinomas (NPC) from Southern China [46]. Finally, two more variants of EBV have been described associated with the LMP-1 gene. A polymorphism of the Xho I restriction site at exon 1 of the LMP-1 gene (position 169425), defines western and Asian strains. The lack Xho I restriction site is common in healthy individuals and EBV-associated diseases in Asia [41], while the presence of the Xho I site is frequently observed in Western countries [47]. In addition, a C-terminal 30-base pair deletion (position 168287–168256) of the LMP-1 gene has been described [48]. However, this variant has not been associated with any specific geographical areas.

### Relevance of EBV strain variations in EBV-associated gastric carcinoma in the Americas

Corvalan and coworkers [31] found that both polymorphisms at the BamHI W1/I1 boundary region (subtypes C and D) and Xho I RFLPs at exon 1 of the LMP-1 gene were present in healthy donors. These authors also identified 2 unique novel recombinant strains, the type C/kept Xho I site and type D/lack of Xho I site [31]. These findings reflect the admixture of different ethnic populations in the Americas [49, 50]. Interestingly, in the case of EBVaGCs, tumors harbor only the western genotype (subtype D and kept Xho I site) (*p <* 0.001). Accordingly, cosegregation of these polymorphisms in EBVaGC was highly significant (OR 16.3 [95% CI 2.5–685]). A consolidated overview of EBV strains in healthy donors and EBVaGC in the Americas is shown in Table 2. Taken together, these findings suggest that EBVaGC in the Americas might be almost exclusively associated to strains from western countries, despite the presence of recombinant strains among healthy donors. Similar findings have been described for *H.pylori* and gastric carcinoma, human papillomavirus and cervical cancer and *M. tuberculosis* and tuberculosis (for a review see [51]. In the case of *H.pylori* and gastric carcinoma, de Sablet et al. [52] identified that strains from the high-risk region were all of European origin, whereas those from the low-risk region were of either European (34%) or African origin (66%). Similar findings have been described for *M. tuberculosis* and human papillomavirus [51] and the concept of “disrupted co-evolution” between the pathogen and its host has been proposed as a contributor for the phylogeographic origin of disease. Here, we propose that this might be the case for EBVaGC, where both Western and Asian wild-type strains and recombinant strains are present in healthy donors. However, only the western wild-type strain is present in gastric cancer. The natural variation of EBV should be further inquired by sequencing a large number of EBV-positive gastric cancer cases and healthy donors.

**Table 2:** EBV strains in healthy donors (HD) and EBV-associated gastric carcinoma in the Americas

		EBNA					F/f variant					type C/D					XhoI kept/loss				
		A		B			F		f			C		D			kept		loss		
References	Country	EBVaGC	HD	EBVaGC	HD	p value	EBVaGC	HD	EBVaGC	HD	*p* value	EBVaGC	HD	EBVaGC	HD	*p* value	EBVaGC	HD	EBVaGC	HD	*p* value
Herrera-Goepfert et al. (2005)	Mexico	5	ND	0	ND	ND	5	ND	0	ND	ND	5	ND	0	ND	ND	1	ND	4	ND	ND
Corvalán et al. (2006)	Colombia	23	159	6	15	0.048176	29	100	0	8	0.046186	2	40	27	116	0.026917	25	115	4	55	0.043103
Ordonez et al. (2011)	Peru	0	ND	ND	ND	ND	ND	ND	ND	ND	ND	5	101	6	17	0.00088	9	17	2	105	0
Corvalán et al. (2006)	Chile	36	98	5	7	0.27439	40	94	0	9	0.012438	0	45	35	54	0	35	19	0	45	0
	Consolidate	64	257	11	22	0.072	74	194	0	17	< 0.001	12	186	68	187	< 0.001	70	151	10	160	< 0.001

### Role of host molecular signaling in the pathogenesis of EBV-associated gastric carcinoma

The TCGA network has proposed a novel molecular classification of gastric carcinoma that recognizes, for the first time, a subtype of tumors positive for Epstein-Barr virus: the EBVaGC. This subtype often displays frequent *PIK3CA* mutations, amplification of *JAK2*, CD274 (also known as PD-L1) and *PDCD1LG2* (also known as PD-L2), as well as unique CpG island methylator phenotype (CIMP) [4]. Although overexpression of PIK3CA has not been significantly associated with any clinicopathological features, loss of *PTEN*, a negative regulator of *PIK3CA*, was independently associated with poor prognosis (*p =* 0.011) [53]. It has been proposed that the PI3K/AKT signaling pathway might also contribute to the switch from latency to the lytic state of the virus, a process known as EBV reactivation [54, 55]. Iwakiri and Takada [56] have shown that PI3K/AKT signal transduction contributes to the transcription of the BZLF1 gene, the master regulator of the lytic cycle and one of the most important genes in the reactivation of the EBV [54, 55]. The main EBV oncoproteins LMP-1 and -2 can activate the PI3K/AKT pathway through the CTAR regions of their transmembrane domains, activating downstream signaling pathways [57]. Polymorphisms of Xho I site at exon 1 of the LMP-1 gene [31] might influence this activation favoring global differences of EBVaGC. Further experiments to confirm this issue are warranted. The JAK/STAT pathway is involved in a range of physiological and cellular processes. *JAK2* gene is amplified and overexpressed in EBVaGCs, but no significant association with survival was found [58]. Administration of JAK2 inhibitors, such as AZD1480, AG-490 and WP-1066, may represent potential therapeutic strategies for EBVaGC [59–61]. Significant amplification of PD-L1 was found in EBVaGC [62]. However, this finding was not associated with any clinicopathological characteristics or survival rates [58]. Interestingly, LMP-1 cooperates with IFN-gamma to increase the expression of PD-L1 and this overexpression is suppressed by knocking down LMP-1 in EBV positive cell lines [63]. One final characteristic of the EBVaGC by the TCGA is the highest frequency of DNA hypermethylation [19]. Evaluation of promoter and non-promoter CpG islands of the human genome has found that EBV infection leads to an extensive methylation of both host and viral genomes, being more extensive than any tumor type from the TCGA network [4, 64, 65]. In particular, hypermethylation of the CDKN2A promoter region has been detected in all studies [10, 19, 66–71]. Whether specific patterns of DNA hypermethylation may be associated to EBVaGC in the Americas is another question which remains to be answered.

### Role of environmental conditions in the pathogenesis of EBV-associated gastric carcinoma

#### Birth order and age

In America, Campos et al. [72] reported that patients born as the eldest child showed the lowest frequency of EBVaGCs (OR = 0.2, 95% CI = 0.1–0.8, *P =* 0.007). These findings were contrary to what has been reported in Asia, where EBVaGC was more frequently observed among eldest child [73]. In America, children got the first EBV infection at a younger age than in eastern populations [74]. This can be an important issue which should be further investigated. A relationship between EBVaGC and onset age for EBV infection has been reported in Mexican-descendants in the U.S, as well as residents from Mexico and Brazil [9, 11, 14]. A proportion of EBVaGC at younger age has also been reported in Colombia and Chile [12, 16]. Further research is required to clarify the relevance of this issue.

### Tobacco and alcohol consumption

In Colombia, Mexico and Honduras, smoking has been associated to the onset of EBVaGC (48%, 43% and 46%, respectively) [11, 75–77]. The association between alcohol consumption and the risk of EBVaGC has been explored in Honduras [76, 77], presenting 43% of association. The relationship between smoking, EBV infection and the risk of developing gastric cancer should be an interesting topic for future investigation. An international case-case comparison of smoking and alcohol consumption associated with EBVaGC risk, including the aforementioned studies [77], indicate that smoking association is stronger for EBV-positive tumors (OR = 1.5 [95% CI 1.01–2.3]). In the case of alcohol consumption, a null association was found [77]. Further research in the Americas will be necessary to better determine these associations.

### Diet

A study conducted in Cali, Colombia reported no associations between EBVaGC and high salt intake or exposure to metallic dust [75]. In addition, no significant associations were found with other dietary habits including salt, fruit and vegetable intake; steamed, fried or smoked foods; or food preparations by baking (oven), barbequing (coal) or roasting [75]. These results are unique to the Americas and contrary to those reported in Asia, where EBVaGC has been associated to metallic dust exposure and salt intake [73]. On the other hand, a study in Korean population reported that daily intake of the mycotoxin aflatoxin B1 (AFB1) was significantly higher among gastric cancer patients when compared with control subjects (1.65 ± 0.72 and 1.91 ± 0.87 ng/Kg/day, respectively, *p <* 0.0001) [78]. In addition, Accardi et al. [79] demonstrated that AFB1 stimulates EBV-mediated B-cell transformation using some *in vitro* and *in vivo* approaches, suggesting that this mycotoxin is a co-factor in EBV-mediated carcinogenesis [79]. Thus, the possibility that AFB1 works as a cofactor in EBVaGC in the Americas warrants additional epidemiological and laboratory research.

### *H.pylori* infection

Infection with *H.pylori* is strongly associated with the progression of the gastric precancerous cascade and the development of intestinal type gastric cancer [80, 81]. Camargo and coworkers [82] have recently compared *H.pylori* serologic profiles of EBV-positive and EBV-negative gastric cancer patients from five countries, including 2 from the Americas (Mexico and Honduras). These authors found a marginal statistical significance of catalase antibodies in the EBVaGC group (OR = 3.15 [95% CI =1.50–6.61]) with no regional differences.

### EBVaGC and lymphoepithelioma-like carcinoma of the stomach

**Figure 3:**
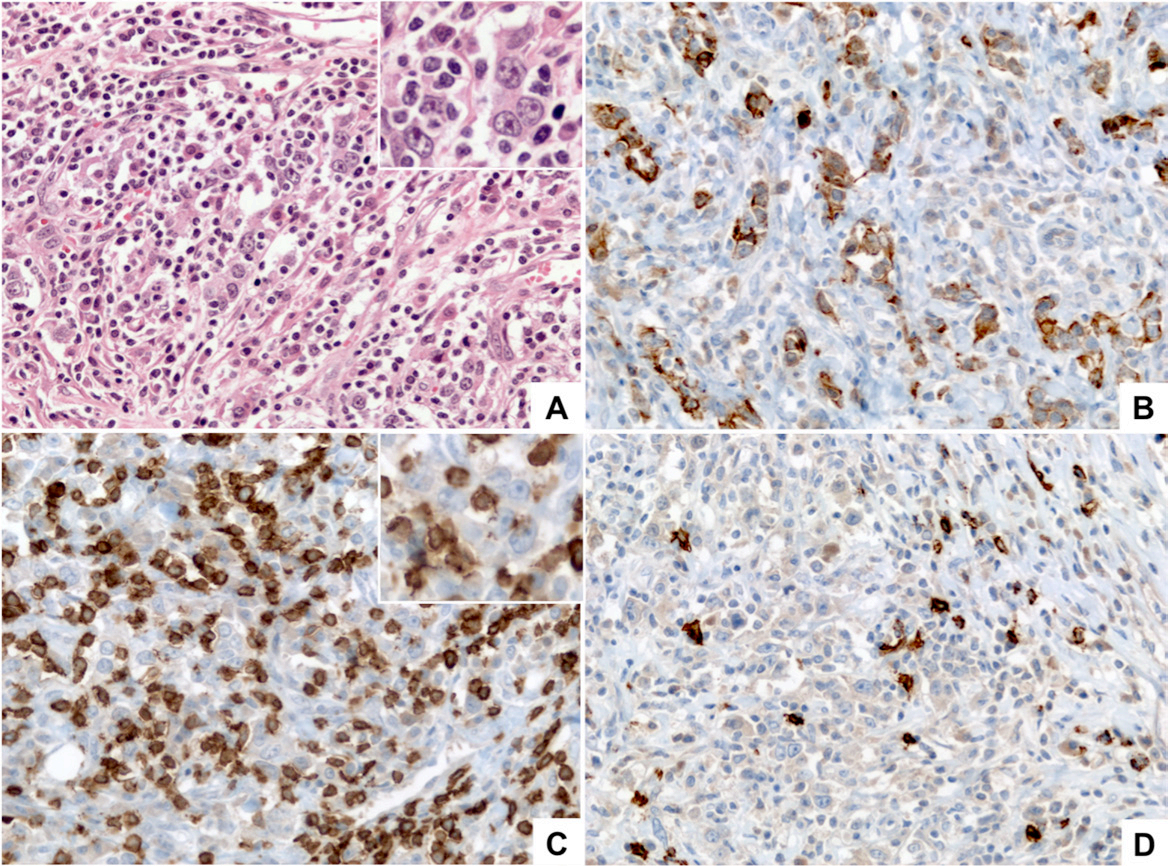
Lymphoepithelioma-like (LEL) carcinoma (**A**) (HE, 400×) shows a poorly differentiated carcinoma formed by medium to large size tumor cells with vesicular nuclei, prominent nucleoli and small amount of eosinophilic cytoplasm (inset) arranged in small clusters and poorly formed cords, intermingled with numerous lymphocytes. (**B**) (Pan-Cytokeratin AE1/AE3, 400×) shows that the tumor cells are positive for cytokeratins demonstrating their epithelial nature and highlighting the poorly formed clusters and cords arrangement. (**C**) (CD3, 400×) highlights the diffuse lymphocytic infiltrate composed mainly by mature T lymphocytes; tumor cells are negative for CD3 (inset). (**D**) (CD20, 400×) highlights scattered B-lymphocytes within the infiltrate.

Watanabe and coworkers [83] described “gastric carcinoma with lymphoid stroma” as a group of tumors showing microalveolar, trabecular, or primitive-tubular patterns with uniformly dense and diffuse lymphoid cell infiltration (Figure 3). This unique phenotype was later defined as lymphoepithelioma-like (LEL) carcinoma and associated with EBV by 3 independent groups [84–86], although it was also recently described as a feature of the MSI subtype of GC [87]. Tumor cells of LEL are immunohistochemically positive for AE3-defined keratin, confirming their epithelial nature. Therefore, the diffuse lymphoid infiltration is a mixture of CD3-positive T cells and CD20-positive B cells (Figure 3). The presence of EBV has been demonstrated in 80%–86% of cases [88, 89]. Retrospective evaluation of a 274-case series from a 14-year period (ordinary gastric carcinoma cases matched by age and sex) showed that LEL tumors had distinct clinicopathologic features and more favorable prognosis [88]. Interestingly, almost half of LEL EBV-positive cases with differentiated components showed the presence of EBV in the non-LEL component as well [88]. Gutierrez and colleagues [90] classified EBV into five subtypes, based on the EBNA-1 sequence in the carboxy-terminal region, which we have named according to the amino acid at position 487 in EBNA-1. Since this amino acid residue was highly predictive of the observed mutation patterns, they termed two of these subtypes as ‘prototypes’ (P-ala and P-thr). On the other hand, they identified 3 other subtypes as “variants” (V-pro, V-leu and V-val), because they differed more markedly from P-ala than does P-thr. According to this EBV classification, Cheng and coworkers [89] compared clinical and molecular features of LEL EBV-positive with EBVaGC and demonstrated that the V-leu and P-ala subtypes were predominant in EBV-positive gastric LEL, which is different from the predominant V-val subtype in ordinary EBVaGC. In addition, EBV-positive gastric LELC has a favorable prognosis when compared to ordinary EBVaGC (median survival time 43.0 vs. 18.0 months, *p* = 0.035). In the Americas, LEL carcinomas have been included as part of an EBVaGC series with prevalences ranging from 1.1% in Chile to 7.6% in Brazil [13, 16]. Whether LEL is a variant of EBVaGC needs to be addressed in future investigations.

## CONCLUSIONS

Novel molecular classifications and meta-analysis have identified EBV as a distinct etiological agent for gastric cancer. An important characteristic of EBVaGC is its higher prevalence in the Americas as compared to Asia. Our meta-analysis estimate a prevalence of 11.4% of EBV infection, more frequently observed in males with predominantly diffuse-type histology. Specific EBV genes such as EBERs, EBNAs, BARTs and LMP are the most actively expressed transcripts. Variations in the sequences of these genes might be associated with the observed phylogeographic diversity of EBV strains across the region. Polymorphisms at the BamHI W1/I1 boundary region and Xho I RFLPs at exon 1 of the LMP-1 gene have been found in healthy donors, reflecting the admixture of different ethnic populations in the Americas. However, this is not the case for gastric cancer, since almost all EBVaGCs harbor exclusively the western genotype (subtype D and kept Xho I site). We propose that “disrupted co-evolution” between EBV and healthy population might contribute to the origin of the disease. Patients born as the eldest child and age of onset of EBV infection are important issues that should be further investigated. Smoking is strong risk factor for EBV-positive tumors, but no associations have been found with dietary habits in the Americas. *H.pylori* infection identified a marginal statistical significance of catalase antibodies in the EBVaGC group. Finally, “gastric carcinomas with lymphoid stroma” described by Watanabe [83] is a common phenotype of MSI and EBV subtypes of GC [87]. LEL has been associated with EBV infection in more than 80% of cases, where most of LEL carcinomas have been included as part of EBVaGC series. Whether LEL is a variant of EBVaGC needs to be addressed in future investigations.

### Search strategy and selection criteria

Data for this Review were identified by searches of PubMed/Scopus/Scielo from relevant articles published between 1959 and September 2016 in English, using the search terms “gastric cancer” and “Epstein-Barr virus” with special emphasis on articles published from the Americas (North, Central and South American countries). Epidemiological data were further searched by GLOBOCAN 2012 [91]. Only studies in which EBV was detected in primary tumors by *in situ* hybridization of EBER were considered. The resulting studies were manually curated according to their relevance to EBVaGC in the Americas and potentially relevant articles were then evaluated in detail. Meta-analyses summarizing frequencies of EBV by EBER in tumor gastric tissues from the Americas including information on the first author, year of publication, tumor location, size of study population and age and sex of the patients (Table 1). Due to the small number of studies available, clinical data were restricted to the variables mentioned above. Meta-analyses were performed by a random-effects model with the Inverse variance method, using the DerSimonian-Laird estimator, a logit transformation, and the Clopper-Pearson confidence interval for individual studies. The results are shown in a log-scale. The I2 statistic, which measures the extent of inconsistency between studies, was assessed. The 95% confidence interval of the odds ratio was used to evaluate differences between groups. Meta-analyses methods are presented in the footnotes of figures and tables.

## DISCLAIMER

The funders had no role in study design, data collection and analysis, decision to publish, or preparation of the paper.
